# Devastating fungal endocarditis involving ascending aorta in a patient with a history of aortic valve replacement: a case report

**DOI:** 10.1186/s13019-024-02733-8

**Published:** 2024-04-08

**Authors:** Parisa Koohsari, Akram Nakhaee, Mehrzad Rahmanian, Faeze Salahshour, Roya Parkhideh, Farnoosh Larti

**Affiliations:** 1 School of Medicine, Tehran University of Medical Sciences, Tehran, Iran; 2 Cardiology Department, Imam Khomeini Hospital Complex, Tehran University of Medical Sciences, Tehran, Iran; 3 Cardiothoracic Surgery Department, Imam Khomeini Hospital Complex, Tehran University of Medical Sciences, Tehran, Iran; 4 Department of Radiology, Advanced Diagnostic and Interventional Radiology Research Center (ADIR), Imam-Khomeini Hospital Complex, Tehran University of Medical Sciences, Tehran, Iran

**Keywords:** *Aspergillus*, Fungal endocarditis, Aortic pseudoaneurysm, Aortic CT angiography, Echocardiography

## Abstract

**Background:**

Fungal endocarditis is a rare but serious condition associated with high mortality rates. Various predisposing factors contribute to its occurrence, such as underlying cardiac abnormalities, cardiac surgeries, prosthetic cardiac devices, and central venous catheters. Diagnosing fungal endocarditis, particularly Aspergillus, poses challenges, often complicated by negative blood cultures.

**Case presentation:**

This report details a case of extensive ascending aorta involvement in Aspergillus endocarditis (AE) in a 24-year-old man with a history of bioprosthesis aortic valve replacement (AVR). Three months post-AVR, he presented with pericardial effusion and aortic rupture, leading to a redo biological valved conduit aortic root replacement (Bentall surgery). Despite the intervention, the tubular graft exhibited extensive Aspergillus involvement, resulting in graft disruption and significant peri-aortic infection. A second redo procedure involving aortic homograft root replacement was performed. Unfortunately, the patient succumbed two days after the surgery.

**Conclusion:**

A combined approach of medical and surgical therapies is recommended to manage fungal endocarditis. Despite efforts, the mortality rate associated with Aspergillus endocarditis remains unacceptably high, with no significant difference observed between combination therapy and antifungal treatment alone. Further research is essential to explore novel therapeutic strategies and improve outcomes for patients with this challenging condition.

**Supplementary Information:**

The online version contains supplementary material available at 10.1186/s13019-024-02733-8.

## Background

Fungal endocarditis (FE) is a rare condition with an overall mortality rate of over 50%. However, the incidence is expected to increase due to advances in medical and surgical therapies, like reconstructive cardiovascular surgery, implantation of intracardiac prosthetic devices, prolonged use of IV catheters, exposure to multiple broad-spectrum antibiotics, and immunosuppression. *Candida* and *Aspergillus* are two common species causing FE. It is shown that *Aspergillus* endocarditis (AE) is related to higher mortality rates than other microorganisms [1]. Herein, we report a rare and fatal AE affecting the ascending aorta in a 24-year-old man, causing multiple complications.

## Case presentation

**Fig. 1:**
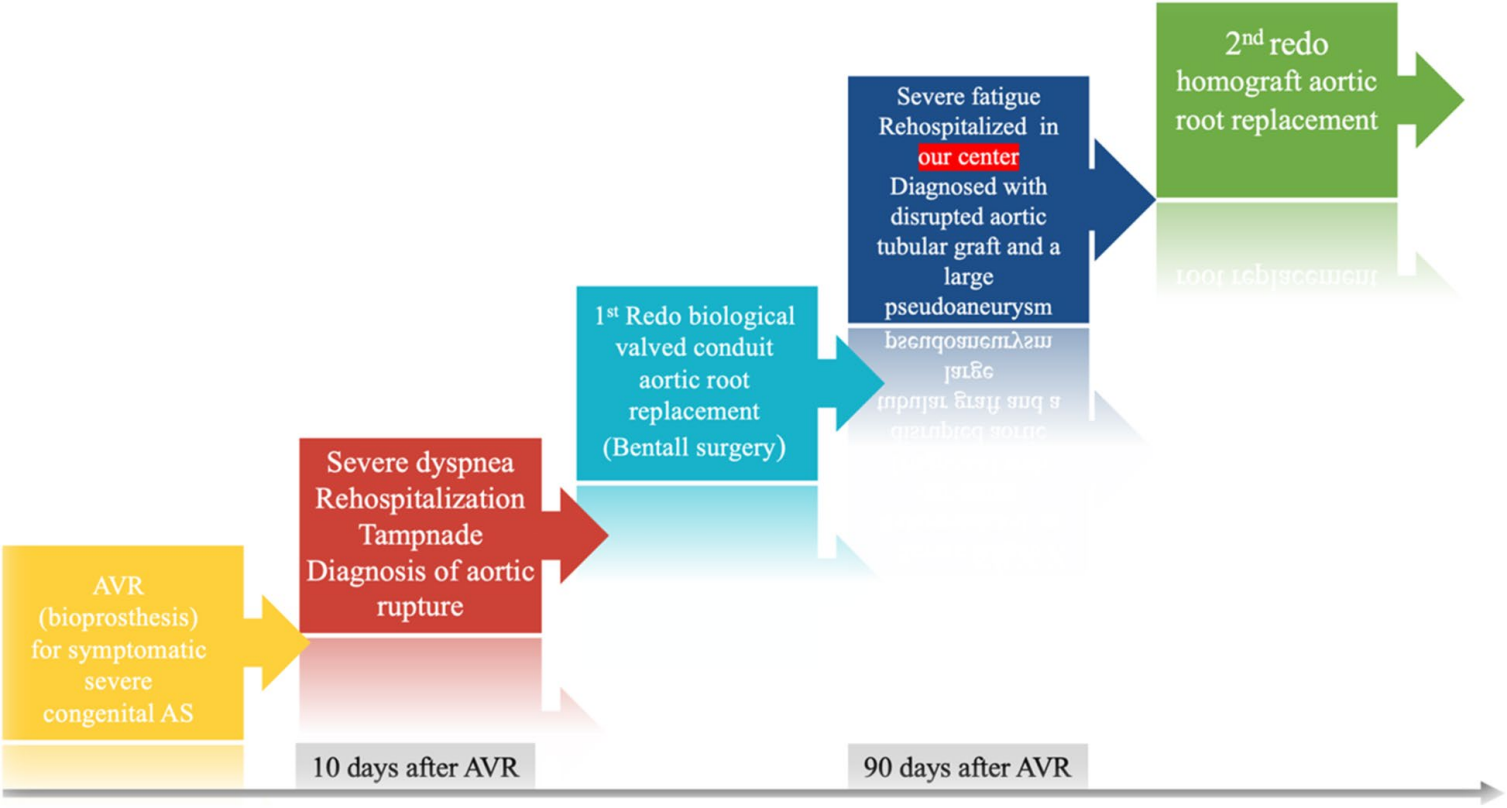
The timeline of the clinical presentation and surgical management

**Fig. 2:**
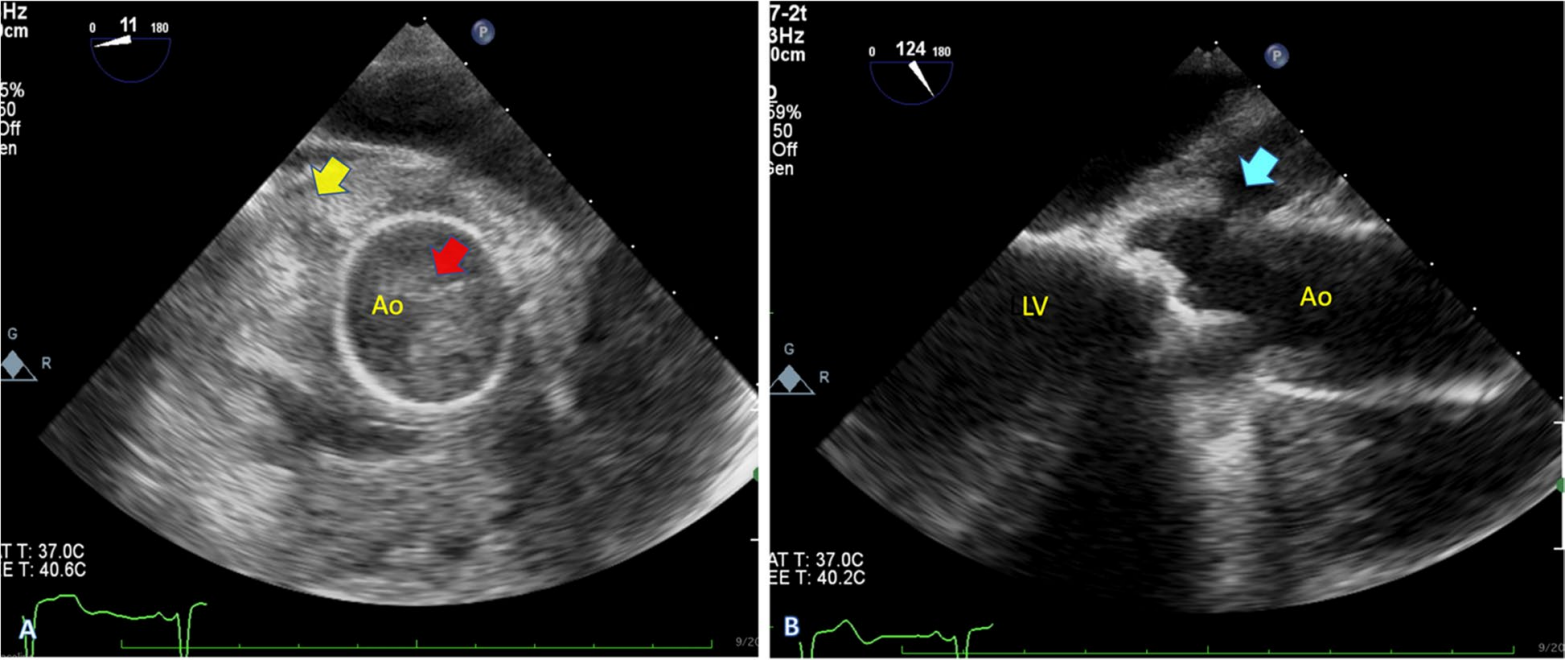
Transesophageal echocardiography: diffuse multiple masses (red arrow) in the tubular aortic graft with extension to the peri-aortic space (yellow arrow), A large disruption (cyan arrow) and pseudoaneurysm in the posterior part of the tubular graft. Ao, Ascending aorta; LV, Left ventricle

**Fig. 3:**
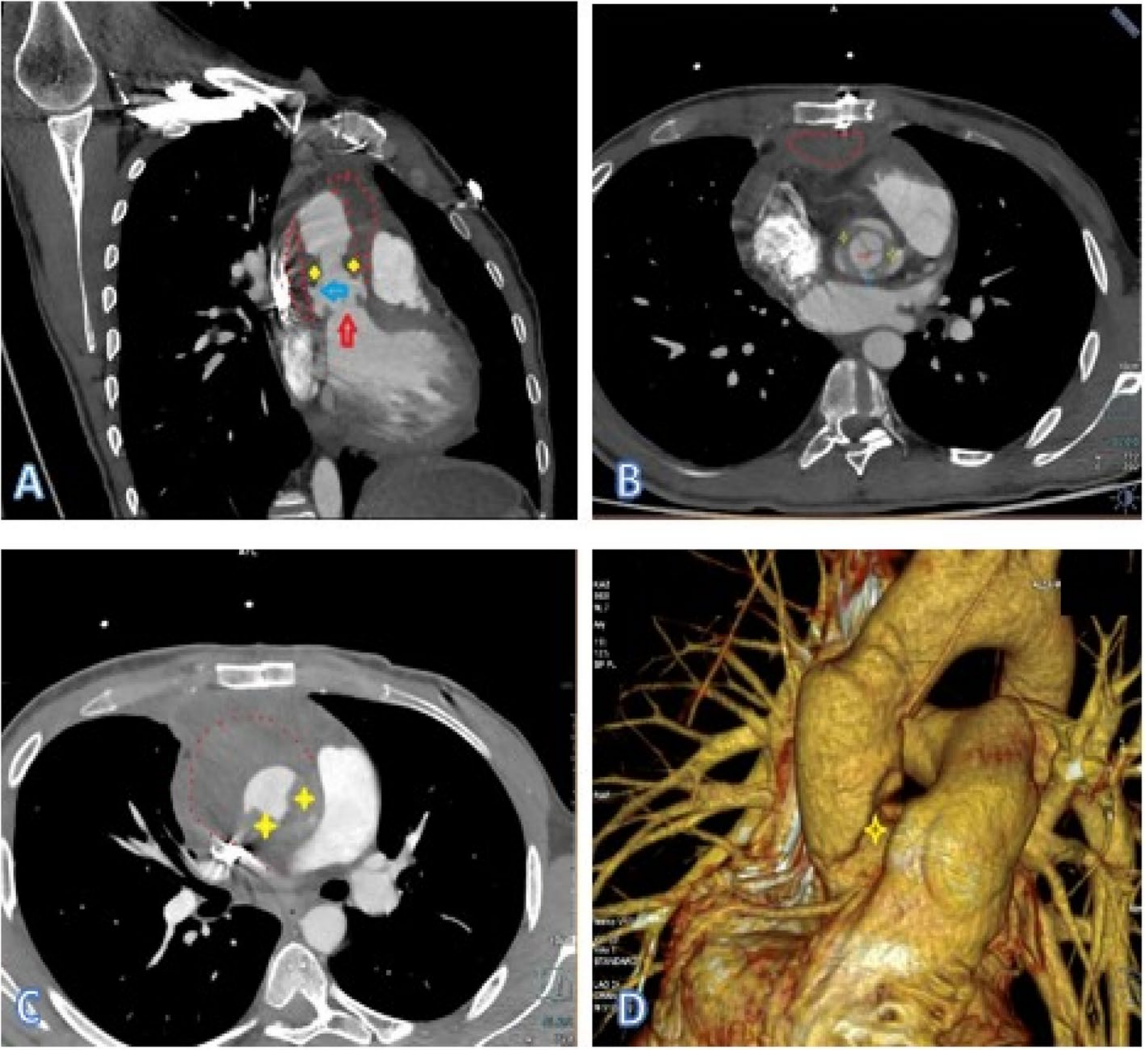
**A**, Oblique coronal reformatted; **B**, axial oblique reformatted at the aortic valve level; **C**, axial oblique reformatted; and **D**, Volume rendering reconstruction of Aortic CT angiography depicts disruption (blue arrow) of aortic root just above the valve (red arrow) along with a large pseudoaneurysm (red dots). An Apple core-like appearance (yellow four-point stars) due to vegetation is seen in the mid-ascending aorta. Most of the pseudoaneurysm is not opacified as it is full of vegetation

A 24-year-old man with a history of aortic surgery was admitted to our hospital with malaise. Three months before admission to our center, he had undergone AVR in another hospital for congenital aortic stenosis. Ten days after discharge, dyspnea urged him to seek medical contact, and he was re-hospitalized in the same hospital. Upon arrival, severe hypotension, massive pericardial effusion, and cardiac tamponade were detected, and he was transferred to the operating room emergently. The diagnosis of aortic rupture due to extensive endocarditis was made in that hospital. Based on the surgical report, the surgeon observed multiple vegetation attached to the aortic valve and ascending aorta. The bioprosthesis was excised, and he underwent a redo biological valved conduit aortic root replacement (Bentall surgery), and antibiotics therapy was started. The exact antimicrobial initiation dosage and timeline were unclear as we gathered these data based on limited discharge notes. Still, the positive results of the vegetation culture changed the treatment regimen to intravenous (IV) anti-fungal. Based on the discharge sheet, he was treated with the proper intravenous (IV) anti-fungal regimen for one month. Then, he was discharged with oral antifungal treatment. Despite two months of therapy with Voriconazole, he continued to suffer extreme fatigue and was referred to our team for further assessment. Figure 1 shows the brief timeline of the disease presentation. After admission to our hospital, transthoracic echocardiography (TTE) followed by transesophageal echocardiography (TEE) was performed. Diffuse multiple masses in the entire tubular graft with significant flow restriction in ascending aorta were detected. A disrupted aortic tubular graft and a large pseudoaneurysm (1.5 cm × 1.8 cm) in its posterior part were seen. Extending this bulky vegetation from the pseudoaneurysm neck to the peri-aortic space created an extraordinary echocardiographic view (Fig. 2, Videos 1, 2, 3 and 4). Following TEE, the patient underwent a contrast-enhanced Computed tomography (CT) scan, which demonstrated polypoid soft tissue densities in the previous surgery site of the ascending aorta in favor of infective vegetations along with contrast media out pouching adjacent to the aortic root indicating pseudoaneurysm (Fig. 3).

Upon admission, three blood culture sets were obtained from separate venipuncture sites before initiating anti-fungal therapy. The intravenous (IV) anti-fungal treatment with Voriconazole and Caspofungin) was restarted. Despite treatment, his symptoms began to worsen during the following three days after admission. He had a fever, malaise, vomiting, and right abdominal, back, and flank pain with right upper quadrant and right costovertebral angle tenderness. The results of three blood cultures were negative. An abdominal ultrasound showed a narrow free fluid rim in Morison’s pouch and splenorenal recess. There was no evidence of appendicitis or hydronephrosis. Increased bladder thickness with debris was seen in favor of pyelonephritis. A spiral abdominopelvic CT scan with oral and intravenous contrast was recommended for evaluating mesenteric ischemia and septic embolism as differential diagnoses. Still, a Doppler ultrasound was performed instead due to acute kidney injury (rise in creatinine level from 1.09 to 1.7 mg/dl). In this situation, a high-risk 2nd redo homograft aortic root replacement surgery was planned. He and his family were informed about the risks and finally underwent surgery. The excised biologic valve was sent for culture, and *Aspergillus flavus* was isolated in the specimen. After surgery, he had acute renal failure and septicemia and received antibiotic and antifungal treatment. Acute tubular necrosis occurred, and he, unfortunately, expired in the intensive care unit (ICU) two days later despite hemodialysis.

## Discussion and Conclusions

Fungal endocarditis (FE) is a rare disorder with increased incidence over the past decades and a more than 50% mortality rate. The most common signs of FE are fever and peripheral embolism, commonly in large brain vessels, mesenteric organs, kidneys, coronary arteries, and the limbs [2]. Several predisposing conditions are identified for FE, including underlying cardiac abnormality and surgery, prosthetic cardiac devices, and central venous catheters as the most common situations [1].

Diagnosing FE is made by following after clinical suspicion: transthoracic or transesophageal echocardiography demonstrating large, bulky vegetations, peripheral blood cultures, histological examination or culture of accessible emboli, and fungal antigen and antibody detection tests. Histologic examination of removed valve vegetations had the highest sensitivity (95%), whereas blood cultures had the least one (54%) [3]. Diagnosing mold-related endocarditis, especially *Aspergillus*, is even more challenging due to more negative blood cultures (81.2% in yeast vs. 30.8% in mold) [1].

Aortic pseudoaneurysm is a rare complication after cardiac surgeries, especially aortic procedures. It is shown that most patients have a history of aortic root replacement or repair (60%) and ascending aorta replacement (32%). Proximal and distal suture lines of implanted valves and aorta were indicated as pseudoaneurysm formation sites in half of the patients [4]. Meshaal et al., in a retrospective cohort study, showed that healthcare-associated endocarditis (HAE), prosthetic valve endocarditis (PVE), and aortic abscess/pseudoaneurysm as strong predictors of *Aspergillus* endocarditis (AE) [5].

Echocardiography is an essential tool in establishing etiological diagnosis. According to European Society of Cardiology guidelines, TTE is recommended as the first-line imaging modality of infective endocarditis (IE). TEE is recommended in patients with prosthetic valves or the presence of a negative or non-diagnostic TTE. The sensitivity of TTE for diagnosing vegetation in IE is approximately 70% in native and 50% in prosthetic valves, compared to 96% and 92% in TEE. Specificity is reported at around 90% for both modalities [6]. Large vegetations are characteristic features of fungal endocarditis, but unfortunately, the rate of false negative echocardiographic results has not been negligible [2]. The role of echocardiography in diagnosing complications after surgeries involving the ascending aorta is vital as the first imaging modality [7].

Multislice CT has a similar accuracy with TEE in detecting vegetations, abscesses, and pseudoaneurysms and is possibly superior in providing information toward the extent and consequences of any perivalvular extension, including the anatomy of pseudoaneurysms, abscesses, and fistulae [8]. Another study demonstrated the usefulness of CT in defining the size, anatomy, and calcification of the aortic valve, root, and ascending aorta in aortic IE, which can be helpful in surgical planning [9].

The aortic valve is the most commonly affected location in FE (62%), followed by the mitral valve (25%) and tricuspid valve (10%). It is shown that the mitral valve's FE is associated with the highest mortality rate either alone or with concomitant aortic valve involvement. On the other hand, tricuspid valve FE had the best survival rate (89%) [3].

## Conclusions

*Aspergillus* is an essential species causing FE. It is shown that the mortality rate in patients with mold endocarditis is significantly higher than in yeast one (82.1% vs. 40.3%) [1].

A combination of medical and surgical therapy is still suggested as the best treatment for FE. Nevertheless, the mortality rate of *Aspergillus* endocarditis is still unacceptably high without a significant difference between combination therapy and antifungal therapy alone [3].

### Supplementary Information

**Additional file 1: Video 1.** Transthoracic long-axis view showed diffuse masses in the aortic tubular graft.

**Additional file 2: Video 2.** Transesophageal view showed a large pseudoaneurysm (1.5 cm × 1.8 cm) in the posterior part of the tubular graft.

**Additional file 3:  Video 3.** Transesophageal view showed a large pseudoaneurysm (1.5 cm × 1.8 cm) in the posterior part of the tubular graft with significant flow restriction.

**Additional file 4: Video 4.** Transesophageal view (short axis of the aortic tubular graft) showed diffuse multiple masses in the tubular graft. Extending these bulky vegetations from the pseudoaneurysm neck to the peri-aortic space created an astonishing echocardiographic view.

## Data Availability

Data is available on reasonable request from the authors.

## Abbreviations

FE: Fungal endocarditis
HAE: Healthcare-associated endocarditis
PVE: Prosthetic valve endocarditis
AE: *Aspergillus* Endocarditis
TEE: TTE (Trans thoracic echocardiography), Transesophageal echocardiography
AVR: Aortic valve replacement
